# Distal forearm fracture risk in a cohort of female and male immigrants and Norwegian-born residents aged 20 to 79 years in Norway

**DOI:** 10.1007/s11657-025-01566-9

**Published:** 2025-06-23

**Authors:** Reyhaneh Lashkari, Cecilie Dahl, Tove T. Borgen, Åshild Bjørnerem, Jan-Erik Gjertsen, Torbjørn Wisløff, Jens-Meinhard Stutzer, Wender Figved, Ann Kristin Hansen, Lene B. Solberg, Frede Frihagen, Espen Heen, Tone K. Omsland

**Affiliations:** 1 Department of Community Medicine and Global Health, Institute of Health and Society, University of Oslo, Pb 1130 Blindern, 0318 Oslo, Norway; 2 Department of Public Health Science, Institute of Health and Society, University of Oslo, Oslo, Norway; 3 Department of Rheumatology, Vestre Viken Hospital Trust, Drammen Hospital, Drammen, Norway; 4 Norwegian Research Centre for Women’s Health, Oslo University Hospital, Oslo, Norway; 5 Department of Clinical Medicine, UiT, The Arctic University of Norway, Tromsø, Norway; 6 Department of Obstetrics and Gynecology, University Hospital of North Norway, Tromsø, Norway; 7 Department of Orthopaedic Surgery, Haukeland University Hospital, Bergen, Norway; 8 Department of Clinical Medicine, University of Bergen, Bergen, Norway; 9 Health Services Research Unit, Akershus University Hospital, Nordbyhagen, Norway; 10 Institute of Clinical Medicine, University of Oslo, N-0407 Oslo, Norway; 11 Department of Orthopedic Surgery, More and Romsdal Health Trust, Molde Hospital, Molde, Norway; 12 Orthopaedic Department, Baerum Hospital, Vestre Viken Hospital Trust, Drammen, Norway; 13 Department of Orthopaedic Surgery, The University Hospital of North Norway, Tromsø, Norway; 14 Division of Orthopaedic Surgery, Oslo University Hospital, Oslo, Norway; 15 Department of Orthopaedic Surgery, Østfold Hospital Trust, N-1712 Grålum, Norway

**Keywords:** Distal forearm fracture, Ethnicity, Immigrants, Season

## Abstract

**Summary:**

The study investigated the risk of distal forearm fractures in adult Norwegian residents according to regions of birth. There were significant differences in fracture risk between the region of birth categories. Although the magnitude of the rates was different between the birth categories, similar sex and seasonal risk patterns were observed.

**Introduction:**

Worldwide, distal forearm fractures (DFFs) are the most common fractures in adults. This study compared incidence rates of first DFFs in women and men in Norway by region of birth, age, and season.

**Methods:**

We included Norwegian residents aged 20 to 79 years with a first DFF between 2010 and 2020 using data from the Norwegian Patient Registry and population estimates from Statistics Norway. Three countries of birth groups were compared: Norwegian-born, Global North (most of Europe, North America, Australia, and New Zealand), and Global South (Asia, Africa, Latin America, Oceania).

**Results:**

Compared to Norwegian-born residents in Norway, immigrants from Global North had 16% and 37% higher age-adjusted DFF incidence rates in women and men, respectively. Compared to Norwegian-born residents, female immigrants from Global South regions had 24% lower rates, whereas male immigrants from Global South regions did not have significantly lower rates. DFF rates were highest in winter for older men and women regardless of birth category, whereas rates in men younger than 50 years were highest during summer months.

**Conclusion:**

We observed significant differences in DFF rates by sex, region of birth, age, and season. Our findings might have important implications for public health efforts and fracture prevention strategies. Nonetheless, further research is necessary to investigate the underlying risk factors and mechanisms driving these differences.

## Introduction

Osteoporosis is a systemic skeletal disease characterized by low bone mass and microstructural deterioration of bone tissue, leading to an increased risk of fracture (1). An ageing population is contributing to an increase in the number of osteoporotic fractures (2, 3). The incidence of osteoporotic fractures is high in Norway (4). A distal forearm fracture (DFF) is one of the most common osteoporotic fractures, and for reasons that are not fully understood, Norway has among the highest reported incidence rates of DFF in the world (5–7). In Europe, osteoporotic fractures account for more disability adjusted life years (DALYs) lost than all common cancers, except for lung cancer (8). Osteoporotic fractures are associated with high morbidity and a high mortality rate (9, 10). A previous DFF increases the risk of a hip fracture (11). Moreover, quality of life after a DFF is reported to be considerably lower compared to the pre-fracture status, especially the first few months following a forearm fracture (12).

The burden of fractures on healthcare resources is growing due to changing population demographics and more complex and costly management strategies (13). Therefore, having an in-depth understanding of fracture epidemiology and possible risk factors is essential for allocating sufficient healthcare resources and for the development of fracture prevention strategies (13). There are significant variations in the incidence of fractures across sex and age (14). DFF rates may vary seasonally due to climate, work and leisure activities, and other factors that change throughout the year such as exposure to daylight (vitamin D production) and indoor temperature increasing the need for clothing (may affect the balance). Studies on DFFs show seasonal variations, peaking in the winter months in adults, especially in women (15–18).

Norway has seen a steep increase in immigration in recent years, now constituting about 17% of immigrants and Norwegian-born to immigrants (19, 20). Globally, the age-standardized rate of osteoporotic fractures is highest in North America and Europe, followed by Asia, the Middle East, Oceania, Latin America, and Africa (21). Asian immigrants in Oslo have a slightly lower risk of DFF than ethnic Norwegians (4). However, the fracture risk within an immigrant group may not accurately represent the fracture risk of their countries of origin due to varying selection mechanisms between groups, and also because they may adapt to a new lifestyle which might also affect fracture risk (22). Individuals migrate due to various reasons such as conflicts, job opportunities, or interpersonal or familial ties, which in turn influence health profiles and associated risk factors of these groups (19). Hence, there can be differences in socio-economic status between different immigrant groups, which might affect fracture risk through previous and current risk factors.

DFF rates in the Scandinavian countries are among the highest in the world, and there is significant seasonal variation in fracture rates (15, 18). However, it remains unclear whether the DFF rates among immigrants are similarly high and show comparable age and seasonal variations to those observed among Norwegian-born residents. This study aimed to compare incidence rates of the first DFF in women and men by region of birth and by age and season.

## Materials and methods

### Definition of outcomes

Data in this study was obtained from the NPR through a larger project called Norwegian Capture the Fracture (NoFRACT) (6). All Norwegian women and men aged 20 to 79 years who received hospital treatment for DFFs between January 1, 2010, and December 31, 2020, were included in the current study. Only the first occurring registration of a DFF diagnosis code was included; later records were excluded. Fractures sustained during 2008 and 2009 were disregarded (wash-out period used to inform coding of first fractures in later years). DFFs coded by the International Classification of Diseases 10th version (ICD-10) as S52.5 (fracture of lower end of radius) and S52.6 (fracture of lower end of both radius and ulna) were included (18). Registrations with ICD-10 codes for follow-up appointments and Nordic Medico-Statistical Committee (NOMESCO) Classification of Surgical Procedures (NCSP) were used to help define incident and prevalent cases (list of codes are previously published (15). In short, records having NCSP codes indicating reoperation were excluded. Except for first-time fracture registrations with a follow-up code after wash-out (3.8% of all remaining records), registrations with ICD-10 codes for follow-up visits were excluded. The rationale for including patients with a first-time follow-up code was that a coverage study from Norway, combining data from NPR and primary health care, found that some patients obtained initial care for their acute fracture in primary care but were shortly after referred to the hospital for surgery or extended care and at that time received a follow-up code in the NPR (23). The validity of forearm fracture diagnoses (all S52, not only distal forearm fractures) in administrative registers and algorithms used to define incident fractures has been investigated and found that the sensitivity of the NPR data from Norwegian hospitals was 90.4% and the positive predictive value was 90.5% (24).

### Person-time at risk

Statistics Norway provided count data on the Norwegian population by 5-year age groups, sex, and country of birth on January 1 from the years 2010 to 2021. Mid-year population estimates, divided by four in the analyses of seasonal variation in rates, were used as substitutes for persons at risk (25). The mid-year population was calculated as the mean of two population sizes for the two following calendar years (the beginning and end of the year for the same 1-year cohorts) as estimated on January 1.

### Exposure variables and stratification factors

The region of birth was stratified into three subgroups:

“Norwegian-born”: Born in Norway, including individuals born to immigrants in Norway.

“Global North”: Immigrants born in the European Union (EU), European Economic Area (EEA) (excluding Norway), Great Britain, Switzerland, the USA, Canada, Australia, and New Zealand.

“Global South”: Immigrants born in Asia with Turkey, Africa, Latin America, Oceania (excluding Australia and New Zealand), and the remaining European countries outside of the EU/EEA.

The data were stratified into four age groups based on the date of hospitalization for the first DFF: 20–34, 35–49, 50–64, and 65–79 years. We excluded those who were 80 years or older, as there were very few immigrants above this age.

### Statistical analyses

The Mann–Whitney *U* test was used to compare age at first DFF between women and men. Sex-specific and age-standardized incidence rates per 100,000 person-years (PY) were calculated by dividing the number of fractures by the person-time at risk (mid-year population estimates). Age-standardization of DFF incidence rates with 95% confidence intervals (CI) was conducted using the direct method (by regional background and season). As a sensitivity analysis, the age-standardized rates by region were recalculated after excluding the age group 65–79 years. The reference population was chosen as the mean age distribution across 2010–2020. Poisson regression analyses adjusted for age were performed to compare incidence rates by region of birth and calculate incidence rate ratios (IRRs). We considered a two-sided *p* ≤ 0.05 to be statistically significant. We defined winter as December, January, and February; spring as March, April, and May; summer as June, July, and August; and fall as September, October, and November. Rates in winter and summer months (separately) were compared to mean rates in the other seasons.

All statistical analyses were performed in Stata (Version 17, Stata Corp LLC, TX, USA). 

### Ethical approval

The study was approved by the Regional Committee for Medical and Health Research Ethics. The University of Oslo performed a Data Protection Impact Assessment in agreement with the General Data Protection Regulation.

## Results

Subjects with missing information on the region of birth were excluded from the analyses, totaling 533 individuals. During 2010–2020 and 40,300,000 PY, the total number of first-occurring DFFs in Norway was 90,297 (65,634 in women and 24,663 in men). A total of 80,420 (89%) of the DFF patients were Norwegian-born; 5730 (6.4%) of the DFF patients were immigrants born in Global North regions, whereas immigrants from Global South regions constituted 4147 DFF patients (4.6%) (Table 1). The median age at first DFF for patients was higher in women than men in all region-of-birth categories (Table 1).

**Table 1:** Total numbers and rates of the first registered distal forearm fracture in Norway 2010–2020, crude and standardized incidence rates stratified by sex and region of birth

		Median age (IQR)^d^	Fractures (*N*)	Population^e^	Crude rate^f^	Stand. rate^f^	CI^h^	IRR^i^
Women	Norwegian-born^a^	62 (17)	59,652	16,900	352	332	330–335	1
	Global North^b^	56 (23)	3323	1213	274	388	374–402	1.16 (1.12–1.20)
	Global South^c^	51 (19)	2659	1726	154	236^ g^	225–247	0.76 (0.73–0.79)
	Total	61 (17)	65,634	19,900	330	325	322–327	
Men	Norwegian-born^a^	53 (26)	20,768	17,200	121	118	116–120	1
	Global North^b^	43 (20)	2407	1712	141	156^ g^	148–163	1.37 (1.30–1.43)
	Global South^c^	42 (21)	1488	1522	98	111	104–118	0.95 (0.90–1.00)
	Total	51 (26)	24,663	20,400	121	121	120–123	

^a^Norwegian-born residents in Norway

^b^Born in EU/EEA countries (except Norway), Great Britain, Switzerland, the USA, Canada, Australia, and New Zealand

^c^Born in Asia, Africa, Latin America, Oceania (excluding Australia and New Zealand), and the remaining European countries outside of the EU/EEA

^d^Interquartile range

^e^In 1000

^f^Per 100,000 person-years

^g^Significantly different from the reference group (Norwegian-born of the same sex) in a Poisson regression adjusted for age

^h^95% confidence interval

^i^Incidence rate ratio adjusted for age

In all ages, IRR of the DFFs was more than twofold higher in women compared to men (overall IRR in women versus men: 2.66, CI 2.63–2.70). Norwegian-born residents’ incidence rates were 332 (CI 330–335) in women and 118 (CI 116–120) in men per 100,000 PY (all age groups) (women to men Norwegian-born residents’ IRR = 2.84, CI 2.80–2.89).

In women aged 50–79 years, the DFF incidence rate was five times higher compared to women younger than 20–49 years (IRR = 5.24, CI 5.14–5.34). The IRR in men aged 50–79 years was 63% higher than in men aged 20–49 years (IRR = 1.63, CI 1.59–1.67).

### Distal forearm fracture incidence rates by region of birth, sex, and age

The age-adjusted IRR in female immigrants from the Global North was 1.16 (CI 1.12–1.20) compared to Norwegian-born residents, and the corresponding IRR in men was 1.37 (CI 1.30–1.43) (Table 1). The incidence rate in women from the Global South was 24% lower compared to the Norwegian-born population (IRR = 0.76; CI 0.73–0.79), whereas no corresponding significant difference was observed in men (IRR = 0.95; CI 0.90–1.00). In sensitivity analyses where we excluded individuals aged 65–79 years, the patterns were nearly identical to those reported in Table 1 (see Supplemental Table 1).

**Fig. 1:**
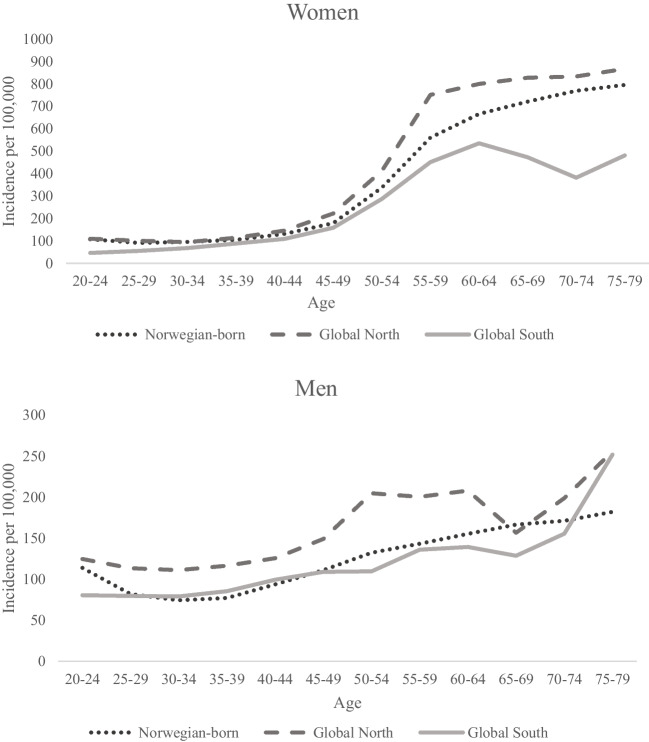
Incidence rates (crude rates) per 100,000 of the first registered distal forearm fracture by age category and region of birth in women and men living in Norway during 2010–2020 (note the use of different scales for the *y*-axes)

In all women, there was a steep increase in the incidence rates of DFF after age 50 which levelled off after age 60 (Fig. 1). Immigrant women born in countries outside the Global North region consistently had the lowest incidence rates across all age groups (*p* < 0.001). The corresponding age patterns in men were less clear, although somewhat higher rates were observed in the youngest (20–24 years) (*p* < 0.001) and oldest (75–79 years) (*p* < 0.001) age groups in all birth region categories (Fig. 1).

### Distal forearm fracture incidence rates by season and age

**Fig. 2:**
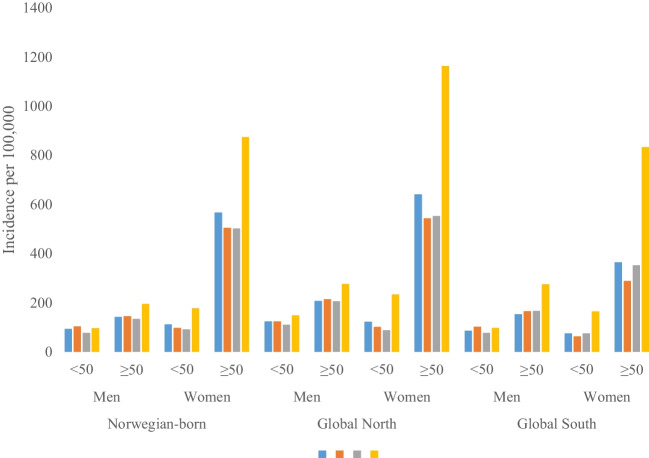
Incidence rates per 100,000 of the first registered distal forearm fracture by season category and region of birth in women and men aged 20 to 49 years and 50 to 79 years living in Norway during 2010–2020

Incidence rates of DFF were significantly higher in winter compared to other seasons regardless of birth region in women (IRR = 1.73, CI 1.71–1.76) and in men (IRR = 1.25, CI 1.22–1.29) (Fig. 2).

Additionally, the higher incidence rates of DFFs in women in winter compared to other seasons did not vary by age: IRR in winter compared to other seasons in female immigrants from the Global North was 2.07 (CI 1.93–2.22), 2.71 (CI 2.52–2.93) in Global South female immigrants, and 1.68 (CI 1.65–1.71) in Norwegian-born women.

Further, the incidence rate of DFF in all men aged 20–49 years was 14% higher in summer compared to other seasons (IRR = 1.14, CI 1.10–1.19). However, in men aged 50–79 years, fracture rates were 41% higher in winter compared to other seasons (IRR = 1.41, CI 1.36–1.46) (Fig. 2).

## Discussion

In the current study, we report age- and sex-specific incidence rates of the first registered DFF by region of birth and season in the Norwegian population aged 20 to 79 years from 2010 to 2020. The study included 90,297 DFFs (40,300,000 person-years) with 9877 DFFs in patients with immigrant background. Irrespective of age, incidence rates of DFFs were higher in immigrants born in the Global North than in Norwegian-born women and men. We also found that in men younger than 50 years, incidence rates of DFF were higher in summer compared to other seasons, regardless of region of birth. This study provides novel insights on differences in fracture risk according to the region of birth in a country with high rates.

In women, the fracture risk patterns by age were observed to be similar irrespective of the birth region; fracture risk increased after 50 years of age and thereafter levelled off after the age of 60 years. In men, fracture risk showed less variation by age, and similar risk patterns according to age were observed in all three countries of birth categories.

In line with a previous Norwegian study on ambient temperature and fractures (18), which found that the highest forearm fracture rate in women > 40 years was around 0 °C, the female age group above 50 years in our study showed noticeably high fracture rates peaking in the winter months, which may indicate an increased risk of falls during icy winter conditions in individuals with osteoporosis. DFF incidence rates in men under 50 years were higher in the summer than in other seasons. This could be due to factors such as a greater number or severity of accidents and falls associated with increased outdoor activities or seasonal work throughout the summer months (26).

Differences in osteoporosis and fracture risk between countries and between different ethnic groups around the world are well described (27–29). For example, in the USA, data from the 1990 s showed that those with African background had a lower rate of DFFs than Caucasians (30). African American women had a higher bone mineral density and lower fracture rates than Caucasian women on average (31, 32). In line with our study, a Swedish study found that fracture rates among male immigrants from Asia and Africa were only slightly lower compared to men born in Sweden (2). However, it should be kept in mind that the quality of the data varies greatly around the world, so the true differences are hard to distinguish (33). Similarly to our study, a study published in 2007 showed that female Asian immigrants in Oslo had a slightly lower risk of a DFF than Norwegian-born individuals (4). The higher rates of DFFs in immigrants from the Global North are supported by findings from a Swedish study where immigrants from Poland, Estonia, and Latvia had significantly higher DFF rates than the Swedish population (2). Findings from the Swedish study support higher DFF rates in specific immigrant groups, such as those from Poland, Estonia, and Latvia, compared to the Swedish-born population, indicating that fracture rates may vary considerably between different countries within larger regional groupings. According to Statistics Norway, there are more than 150,000 immigrant commuter workers in Norway (34). Many of them have seasonal work, and the majority come from countries in Eastern Europe. Further studies should focus on identifying high-risk groups for forearm fracture, both considering country background, occupation, sex, and seasonal differences.

Our findings indicate that immigrants from the Global North had higher incidence rates of fractures compared to Norwegian-born individuals, particularly men. A notable sex difference in fracture risk was observed among immigrants from the Global North, with men showing a stronger relative risk compared to Norwegian-born residents (IRR = 1.37), whereas the increase in women was more modest (IRR = 1.16). Several factors may contribute to this pattern. Male immigrants may face greater changes in occupational roles and physical activity levels after migration, potentially leading to an increased risk of falls and fractures. A significant number of immigrants from the EU come to work in industries such as construction, fishing, and agriculture which often involve high-risk activities that increase the likelihood of fractures; this is particularly true for immigrants from Eastern Europe (19). Furthermore, osteoporosis is often underdiagnosed and undertreated in men, despite comparable morbidity from fractures (35). In contrast, the lower fracture incidence among immigrant women from Global South countries may be explained by cultural and behavioral factors, such as less exposure to slippery outdoor environments in winter (spending more time indoors), stronger family support, or differences in body composition (36, 37). These protective factors might not apply equally to men from the same regions, possibly contributing to the observed sex-specific trends.

Fracture risk in immigrant groups may not accurately reflect that of their home countries due to various migration-related factors (19). Individuals migrate due to conflicts, job prospects, or personal relations, which might indirectly impact the health status of this group. While genetic factors are important, other factors like lifestyle, diet (including calcium and vitamin D intake), physical activity, and access to healthcare are also important. Immigrants from Global North regions might experience different health behaviors, living or working conditions compared to both Norwegian-born residents and other migrant groups, which may affect fracture risk (19). Immigrants may also adapt to the Norwegian lifestyle and over time get an increased fracture risk (22), or experience difficulties adjusting to the winter climate (walking habits, footwear choices, etc.).

Based on our findings, future studies mapping risk factors in subgroups of immigrants with high fracture risk are warranted. Public health initiatives aimed at preventing fractures should be tailored to the specific needs of immigrant populations. For women from the Global North, this could include targeted osteoporosis screening, culturally appropriate education on fall prevention, and increased access to preventive healthcare services.

## Strengths and limitations

The use of data from NPR enables access to large data sets including all Norwegian hospitals. However, the NPR data are registered for administrative purposes, which might have implications for the validity of the data. On the other hand, an algorithm for defining the outcome has been developed and found to have high validity (24). We did not have the exact time at risk measured in days, but we used mid-year population estimates from Statistics Norway. These estimations are appropriate estimates for time at risk, although less precise in the older age groups than in younger ones due to a higher mortality.

Another weakness is that we might have missed fractures sustained abroad by travellers, especially for immigrants who may travel more and perhaps also for longer periods than Norwegian-born citizens. This might, at least partly, explain the lower rates in female immigrants from the Global South. But fractures sustained while travelling might be registered as a follow-up when back in Norway for treatment, and therefore, we are not likely to have missed all fractures sustained abroad. According to Statistics Norway, immigrants are less likely to live in rural municipalities, implying that immigrants might be treated for fractures less often in primary care than Norwegian-born citizens and thus are more likely to be captured by the NPR. A Norwegian study suggests that immigrants in general use primary health care services less often than Norwegian-born individuals, but that the use tends to increase with increased length of stay (38). Moreover, another study found that immigrants from low- and middle-income countries used emergency care more often than natives which also may imply that fractures in immigrants are more often reported to the NPR compared to fractures in Norwegian-born individuals (39). With the universal health care system in Norway, almost all fracture patients are treated at public hospitals and thereby recorded in the Norwegian Patient Registry (NPR), leaving a proportion of uncomplicated forearm fractures (coded by the less specific ICPC-2 system) to the primary health care system in areas located far from hospitals. Consequently, these are not captured in the NPR, but this constitutes a very small proportion of all fractures (23).

In summary, the different biases affecting registrations of DFFs in Norwegian-born individuals and immigrants act in different directions. The higher incidences of immigrants from the EU, North America, Australia, and New Zealand are not likely to be explained by differences in registration. To what extent the lower incidence rates in immigrants in the Global South category are explained by travelling needs to be investigated in future studies.

A notable limitation of our study is the lack of data on ethnicity in the Norwegian register, and due to very low numbers of immigrants from many countries, we were not able to calculate meaningful country-specific estimates of DFF rates. Additionally, the grouping of immigrants based on political and geographical borders, such as European EU versus non-EU countries, might not fully capture the diverse socio-economic and cultural differences within these groups. Future research could explore more detailed subgroup analyses to understand the impact of specific countries or regions on fracture rates.

As shown by the median ages of the different region-of-birth categories, the age distribution of the immigrants is very different from the age distribution of the Norwegian-born residents. Still, DFF rates in immigrants below 50 years tend to be as high as in Norwegian-born individuals. Excluding the age group 65–79 years showed similar differences between the regions, indicating that our results are robust. Future studies could enlighten whether this pattern continues into older age as the immigrant populations grow older and larger.

## Conclusion

Women and men from the Global North had 16% and 37% higher age-adjusted DFF rates than Norwegian-born residents, respectively. Female immigrants from the Global South had 24% lower rates than Norwegian-born residents, but no difference was found in men. In all ages, the DFF rates were about twice as high in women compared to men, regardless of regions of birth categories. Although there were differences in the magnitude of the rates between the region of birth categories, the DFF age patterns showed similar variation in both sexes. In women, the rates of DFFs were higher in winter compared to the rest of the year whereas the age and seasonal variation was less apparent in men. These differences highlight the importance of considering country background and possible occupational exposures in fracture prevention programs. Future studies should focus on mapping risk factors in immigrant groups with high risk, which can be targeted in prevention programs.

## Data Availability

Due to the protection of privacy under General Data Protection Regulation (GDPR) and the Norwegian law, the individual-level data can only be made available after approval by the Regional Committee for Medical and Health Research Ethics.
